# Molecular mechanism of pH sensing and activation in GPR4 reveals proton-mediated GPCR signaling

**DOI:** 10.1038/s41421-025-00807-y

**Published:** 2025-06-25

**Authors:** Chongzhao You, Shimeng Guo, Tianwei Zhang, Xinheng He, Tianyu Gao, Wenwen Xin, Zining Zhu, Yujie Lu, Youwei Xu, Zhen Li, Yumu Zhang, Xi Cheng, Yi Jiang, Xin Xie, H. Eric Xu

**Affiliations:** 1 The State Key Laboratory of Drug Research, Shanghai Institute of Materia Medica, Chinese Academy of Sciences, Shanghai, China; 2 University of Chinese Academy of Sciences, Beijing, China; 3 School of Life Science and Technology, ShanghaiTech University, Shanghai, China; 4 Lingang Laboratory, Shanghai, China; 5 School of Pharmaceutical Science and Technology, Hangzhou Institute for Advanced Study, University of Chinese Academy of Sciences, Hangzhou, Zhejiang China; 6 School of Chinese Materia Medica, Nanjing University of Chinese Medicine, Nanjing, Jiangsu China; 7 Shandong Laboratory of Yantai Drug Discovery, Bohai Rim Advanced Research Institute for Drug Discovery, Yantai, Shandong China

**Keywords:** Cryoelectron microscopy, Extracellular signalling molecules

## Abstract

Maintaining pH homeostasis is critical for cellular function across all living organisms. Proton-sensing G protein-coupled receptors (GPCRs), particularly GPR4, play a pivotal role in cellular responses to pH changes. Yet, the molecular mechanisms underlying their proton sensing and activation remain incompletely understood. Here we present high-resolution cryo-electron microscopy structures of GPR4 in complex with G proteins under physiological and acidic pH conditions. Our structures reveal an intricate proton-sensing mechanism driven by a sophisticated histidine network in the receptor’s extracellular domain. Upon protonation of key histidines under acidic conditions, a remarkable conformational cascade is initiated, propagating from the extracellular region to the intracellular G protein-coupling interface. This dynamic process involves precise transmembrane helix rearrangements and conformational shifts of conserved motifs, mediated by strategically positioned water molecules. Notably, we discovered a bound bioactive lipid, lysophosphatidylcholine, which has positive allosteric effects on GPR4 activation. These findings provide a comprehensive framework for understanding proton sensing in GPCRs and the interplay between pH sensing and lipid regulation, offering insights into cellular pH homeostasis and potential therapies for pH-related disorders.

## Introduction

Maintaining physiological pH homeostasis within the narrow range of 7.35–7.45, is fundamental to cellular function and organismal survival^1–4^. Disruption of this delicate balance occurs in numerous pathological conditions, including diabetes, renal dysfunction, respiratory disorders, and cancer, as well as during intense physical activity and dietary changes^1,5–10^. The human body employs sophisticated regulatory mechanisms to maintain pH homeostasis, encompassing buffer systems, respiratory and renal regulation, and cellular ion exchange^1,5,11^. At the molecular level, three distinct classes of pH sensors orchestrate cellular responses: proton-sensing G protein-coupled receptors (GPCRs), proton transporters (H^+^-ATPases), and acid-sensing ion channels (ASICs)^12–16^. While the structures and activation mechanisms of H^+^-ATPases and ASICs have been extensively characterized^16–18^, the molecular basis of proton sensing by GPCRs remains unclear.

GPR4, a prominent member of proton-sensing GPCRs, commands particular attention due to its ubiquitous expression and crucial roles in endothelial function, tumor biology, and metabolic acidosis regulation^7,19–24^. It maintains partial activity at physiological pH while achieving full activation under acidic conditions^25,26^. Although GPR4 predominantly signals through G_s_ proteins, it exhibits remarkable coupling plasticity across G protein subtypes, with the G_q_ pathway demonstrating a distinct pH-sensing range that potentially suggests an alternative sensing mechanism^27^. Two prevailing theories have emerged to explain the proton-sensing mechanism of GPR4. One is centered on extracellular histidine residues^25,28–30^, and another focuses on p*K*_a_ shifts in buried acidic residues^26,31,32^. Recent evolutionary analysis has highlighted the critical role of the extracellular domain (ECD) of non-human GPR4 in proton sensing^33^, while a systematic functional study of proton-sensing receptors, particularly GPR68, has provided valuable insights into pH sensing mechanisms^33,34^. However, the precise molecular mechanism of human GPR4 proton sensing remains to be fully elucidated.

A fascinating aspect of GPR4 regulation involves lysophosphatidylcholine (LPC), an abundant plasma membrane lipid^35,36^. LPC is associated with endothelial functions and immune regulation, while serving as a precursor for bioactive lipids through autotaxin-mediated conversion^36–38^. Evidence suggests that LPC modulates GPR4 activity and potentially mediates both lysophospholipid-dependent and -independent pathways in tumor development^35,36,38^. However, the molecular basis of LPC–GPR4 interactions and their physiological significance remains poorly understood.

The clinical relevance of GPR4 stems from its pivotal role in endothelial cell regulation and its implications in various pathological conditions^7,19,20,39–41^. Under acidic conditions, GPR4 activation promotes angiogenesis and enhances vascular permeability, potentially facilitating tumor growth and metastasis while perpetuating acidosis^10,19,23,39,42^. These findings have established GPR4 as a promising therapeutic target, spurring efforts to develop selective antagonists^43–46^.

To address the critical need for mechanistic understanding in drug development, we present high-resolution structures of GPR4 in complex with G_s_ and G_q_ transducers under physiological (pH 7.4) and acidic (pH 6.5) conditions. Our structural analysis reveals critical insights into proton sensing and activation mechanisms. Furthermore, we identify and characterize LPC-binding sites, illuminating their functional impact on GPR4 activation. Through comprehensive structural analysis, pharmacological profiling, mutagenesis, and computational approaches, we provide a detailed molecular framework of GPR4’s proton-sensing mechanism and lipid interactions. These findings not only advance our knowledge of pH-sensing GPCRs but also establish a foundation for structure-based drug design targeting pH-related pathologies.

## Results

### Unique conformations of GPR4 complexes

**Fig. 1:**
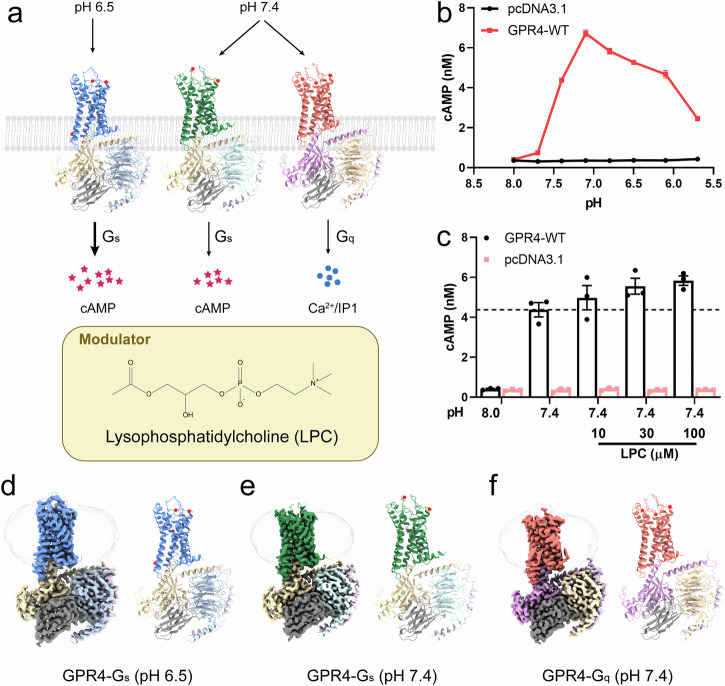
Overall structures of GPR4–G_s_/G_q_ complexes at acidic and physiological pH conditions. **a** Schema diagram of GPR4 activation of G_s_/G_q_ pathways at various pH conditions. **b** Curves showing pH-dependent cAMP accumulation in cells overexpressing GPR4 or pcDNA3.1. **c** LPC-induced activation of GPR4 at pH 7.4 measured in a dose-dependent manner using a cAMP accumulation assay. Values are represented as means ± SEM of three independent experiments (*n* = 3). **d**–**f** Overall structures and EM-density maps for GPR4 complexes. The receptors are colored blue (GPR4–G_s_ at pH 6.5), green (GPR4–G_s_ at pH 7.4), and salmon (GPR4–G_q_ at pH 7.4), respectively. Gα_s_ subunit is colored wheat and Gα_q_ subunit is colored purple. Colors of the Gβγ subunits are shown as indicated. Red balls refer to water molecules in our structures.

GPR4 exhibits a distinct pH-dependent activation profile, responding to pH changes between 5.8 and 7.8, with inactivation occurring above pH 7.8, as confirmed by cAMP accumulation assays^27^ (Fig. 1a, b). To elucidate the molecular mechanisms of proton sensing and activation in GPR4, we determined high-resolution cryo-EM structures of GPR4 in complex with G_s_ under both physiological (pH 7.4) and acidic (pH 6.5) conditions at resolutions of 2.59 Å and 2.36 Å, respectively. We also determined the cryo-EM structure of GPR4 in complex with G_q_ at a resolution of 2.55 Å at pH 7.4 (Fig. 1d–f; Supplementary Figs. S1–S3 and Table S1). Our density maps enabled precise modeling of the receptor, G proteins, and associated components (Supplementary Fig. S4).

**Fig. 2:**
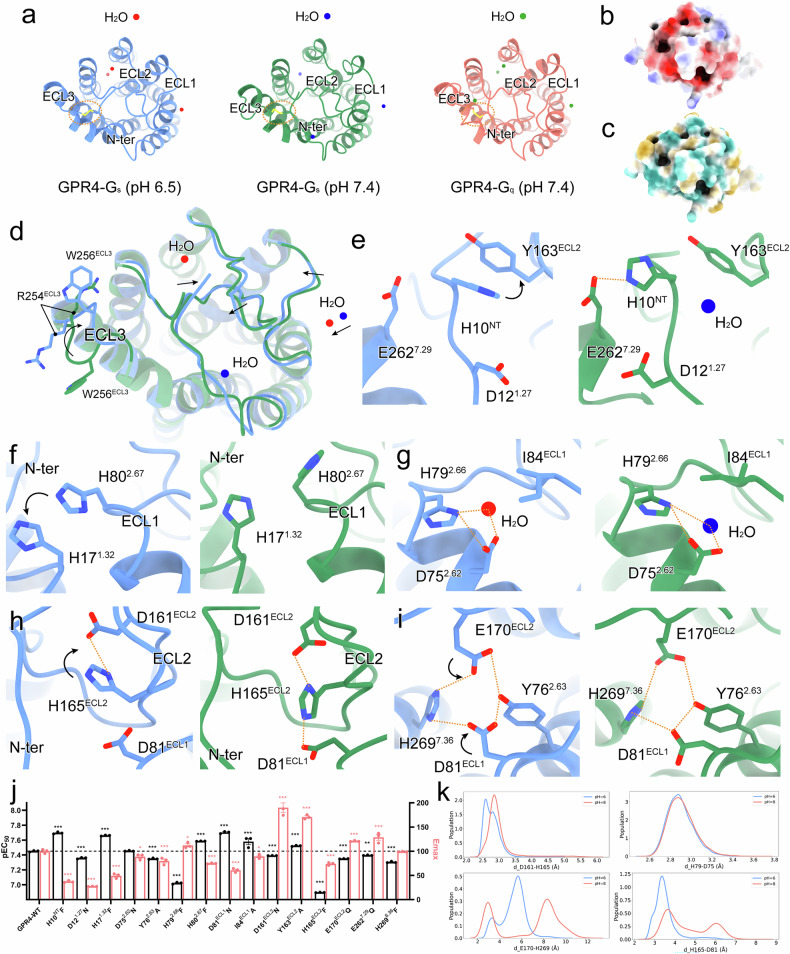
Proton recognition mode of GPR4. **a** The top view of active GPR4 with the distribution of protons around ECD. The disulfide bonds are labeled by orange dashed circles and the water molecules are displayed as spheres. Colors are shown as indicated. **b**, **c** The surface of ECD in the GPR4–G_s_ complex at pH 6.5 by electrostatic (**b**) and hydrophobic (**c**) analyses. **d** The structural superposition of ECD in the GPR4–G_s_ complexes at pH 6.5 and pH 7.4. **e**–**i** Detailed interactions and comparisons of GPR4 at pH 6.5 and pH 7.4. The displacement of residues and polar interactions are marked by black arrows and orange dashed lines, respectively. **j** Effects of GPR4 mutations on the potency of pH-induced cAMP accumulation. The black bars represent the pEC_50_ values of pH-induced responses in wild-type GPR4 (WT) and mutants, while the red bars indicate the maximum cAMP concentrations induced by pH in GPR4 WT and mutants, both normalized to WT. A decrease in pEC_50_ indicates reduced sensitivity to pH. The original data are provided in Supplementary Fig. S9. Values are shown as means ± SEM from three independent experiments. *P < 0.05, **P < 0.01 and ***P < 0.001 by one-way ANOVA followed by multiple comparison test, compared with WT. **k** The extracellular conformation distribution under pH 6.0 and pH 8.0. The side-chain minimal distance distribution of D161^ECL2^–H165^ECL2^, D75^2.62^–H79^2.66^, E170^ECL2^–H269^7.36^, and D81^ECL1^–H165^ECL2^.

A defining feature of active GPR4 structures is the cooperative organization of ECD. Specifically, the N-terminus and TM1 exhibit a distinctive bend toward the central region of the receptor, while extracellular loops (ECLs) form stabilizing interactions with the N-terminus through an intricate network of water molecules (Fig. 2a). Surface analysis reveals a highly acidic and hydrophilic ECD, a characteristic crucial for proton sensing^34^ (Fig. 2b, c). The high resolution of our structures (2.36–2.59 Å) allows precise mapping of these water molecules, providing important insights into their role in proton sensing and signaling (Supplementary Fig. S4d).

Our structures reveal several unique architectural features that distinguish GPR4 from typical class A GPCRs (Supplementary Fig. S5). First, the bent TM1 forms extensive networks with ECLs, creating a compact structure essential for proton sensing (Fig. 2a; Supplementary Fig. S5). The structures exhibit hallmark features of GPCR activation, including pronounced outward movement of TM6 at the cytoplasmic end (Supplementary Fig. S5a). However, GPR4 displays distinctive characteristics shared with other pH-sensing receptors like GPR65 and GPR68^34^ (Supplementary Fig. S5f). These include a unique two-helical-turn extension of TM7 at the extracellular side, enabling ECL3–N-terminus interaction; inward movement of both the N-terminus and the extracellular end of TM1, reminiscent of lipid-liganded GPCRs like GPR3, GPR40, and GPR119^47–50^; and an exceptionally compact ECD architecture where the N-terminus coordinates ECL1–3 interactions, crucial for proton sensing^33,34^ (Supplementary Fig. S5b–e). Second, a rare disulfide bond between C9^NT^ and C258^7.25^, also present in GPR68^34^, appears to be a specialized feature of proton-sensing GPCRs (Fig. 2a; Supplementary Fig. S5f). Mutation of either cysteine significantly impairs both proton sensitivity and receptor activation, highlighting the critical role of this structural element in receptor function^34^ (Supplementary Fig. S6).

Notably, our density maps revealed multiple associated lipids, including cholesterol and phospholipids (Supplementary Fig. S4). Of particular interest, we identified a clear density corresponding to LPC, previously proposed as an endogenous GPR4 ligand. Functional validation through cAMP accumulation assays demonstrated that LPC acts as a positive allosteric modulator of GPR4 in a dose-dependent manner (Fig. 1c). This finding aligns with recent reports of LPC’s role in modulating ADGRF1^51^ and GPR119^47^, suggesting a broader significance of LPC in GPCR signaling.

### The proton recognition mechanism of GPR4 complexes

Among proton-sensing GPCRs, GPR4 stands out due to its uniquely high histidine content (Supplementary Fig. S7) and distinctive ability to maintain activation at elevated pH ranges (7.4–7.8)^30^. The imidazole side chain of histidine serves as a precise proton receptor through its pH-dependent charge distribution^52–54^, while aspartic and glutamic acid residues contribute to proton sensing through electrostatic interactions^33,34,55,56^. The enrichment of both histidine and acidic amino acids in ECD of proton-sensing GPCRs suggests their orchestrated role in proton detection (Supplementary Fig. S7).

Our high-quality structures reveal an extended polar network within ECD, comprising numerous proton-titratable residues, particularly in the N-terminus and ECL2. Water molecules are clearly visible throughout the GPR4 ECD (Fig. 2a–c). While most of the ECD maintains similar conformations at pH 6.5 and 7.4, ECL3 exhibits distinct rotational changes (Fig. 2d). The complex at lower pH demonstrates tighter ECD association, accompanied by rotational shifts in R254^ECL3^ and W256^ECL3^ (Fig. 2d).

GPR4 exhibited enhanced agonist activity at pH 6.5 vs pH 7.4 (Fig. 1b). Comparative analysis of the G_s_-coupled GPR4 complexes at pH 6.5 and pH 7.4 reveals key conformational changes underlying proton sensing. At pH 6.5, the protonated H10^NT^ rotates toward Y163^ECL2^ to form a π–π interaction, while at pH 7.4, it interacts with E262^7.29^ and a water molecule that restrains its movement (Fig. 2e). The protonated H17^1.27^ and H80^2.67^ establish a stable π–π interaction at pH 6.5, maintaining N-terminus–ECL1 connectivity (Fig. 2f). A conserved water molecule occupies the cavity formed by D75^2.62^, H79^2.66^, and I84^ECL1^ at both pH conditions, creating a local polar network that stabilizes ECL1 conformation (Fig. 2g). The water molecule shifts slightly inward with H79^2.66^ rotation, triggering conformational changes (Fig. 2d, g). H165^ECL2^ undergoes inward rotation to coordinate with D161^ECL2^, forming strong electrostatic interactions (Fig. 2h). Additionally, Y76^2.63^, D81^ECL1^, and E170^ECL2^ generate a polar network that coordinates with the protonated H269^7.36^ (Fig. 2i). The evolutionarily conserved π–π stacking between H155^4.63^ and W177^5.34^ in non-human GPR4^33^ is preserved in our structures, and mutations disrupting this interaction significantly reduce receptor activity (Supplementary Fig. S8). The pH-dependent conformational changes highlight the crucial role of these residues in proton sensing (Fig. 2j; Supplementary Fig. S9). At pH 7.4, GPR4 maintains certain features, including interactions stabilizing the conformation of the N-terminus and ECLs (Fig. 2d–i). Structure-based p*K*_a_ calculations identify H269^7.36^ as the primary protonated histidine at pH 6.5 (Supplementary Fig. S10), which is supported by molecular dynamics (MD) simulations analyzing specific residue side-chain minimal distance distributions (Fig. 2k).

**Fig. 3:**
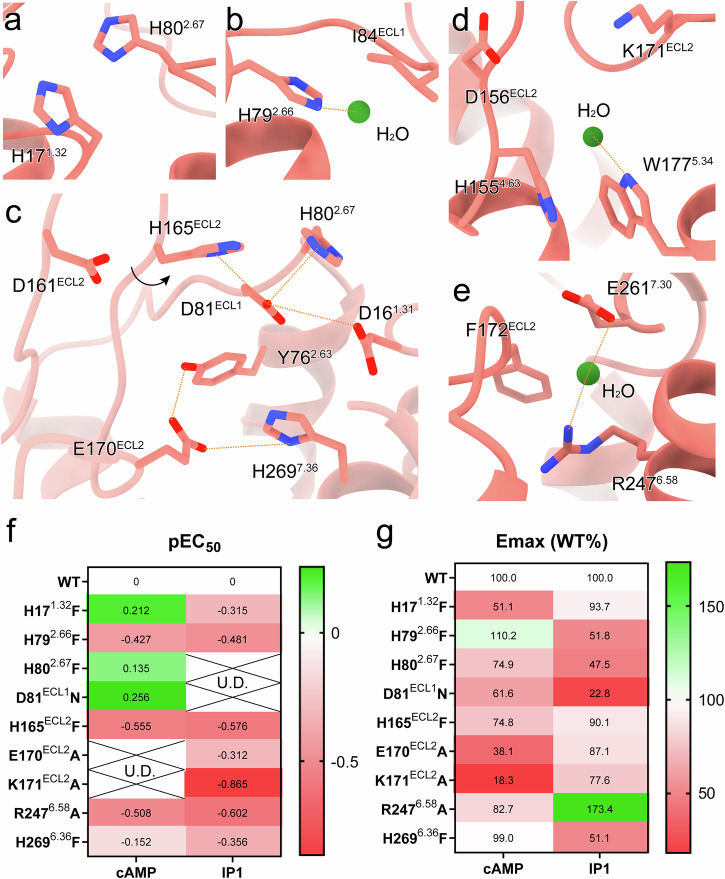
Proton recognition mode of GPR4–G_q_ complex at physiological pH. **a** The preserved interaction between H17^1.32^ and H80^2.67^. **b** The interaction between H79^2.66^ and the common water molecule. **c** The extended polar network of H165^ECL2^ and H269^7.36^. The rotational direction of residues is labeled by a black arrow indicating the rotation of H165^ECL2^, compared with H165^ECL2^ in the GPR4–G_s_ complexes, and polar interactions are shown as orange dashed lines. **d**, **e** The special networks involving the water molecules in G_q_-coupled GPR4. The water molecules are displayed as green spheres. The polar interactions are shown as orange dashed lines. **f**, **g** Effects of mutations on cAMP and IP1 accumulation responses. ΔpEC_50_ represents the difference between pEC_50_ values of GPR4 WT and mutants. U.D. means undetectable because the maximum activation level is below 50% to determine pEC_50_ values (**f**). E_max_ values represent the maximum cAMP or IP1 accumulation induced by various pH conditions in GPR4 WT and mutants, which are normalized to WT (**g**). Heat map is generated on the basis of the pEC_50_ or E_max_. Values are shown as means of three independent experiments. The original data are provided in Supplementary Fig. S11.

The G_q_-coupled GPR4 at pH 7.4 shares conformational similarities with G_s_-coupled GPR4 complexes (Fig. 2a) while exhibiting distinct features. The H17^1.27^–H80^2.67^ π–π interaction and H79^2.66^–I84^ECL1^–water molecule network are preserved (Fig. 3a, b). However, the H165^ECL2^–H269^7.36^ polar network undergoes expansion (Fig. 3c). D81^ECL1^ adopts a unique conformation, interacting with H80^2.67^ and H165^ECL2^ to create an enhanced polar environment (Fig. 3c). The D161^ECL2^–H165^ECL2^ interaction maintains its proton-sensing role but with an increased distance (Fig. 3c). The D16^1.31^–E170^ECL2^–H269^7.36^ network shows tighter association compared to that in the G_s_-coupled GPR4 at pH 7.4 (Fig. 3c). Through functional mutagenesis studies, we observed that H80^2.67^F and D81^ECL1^N mutations significantly reduced G_q_-IP1 signaling, whereas E170^ECL2^A and K171^ECL2^A mutations resulted in a more significantly reduced maximal response in G_s_-cAMP signaling (Fig. 3f, g; Supplementary Fig. S11). The GPR4–G_q_ complex also displays distinct water molecule networks (Fig. 3d, e), suggesting G protein-specific diversity in proton-sensing mechanisms.

**Fig. 4:**
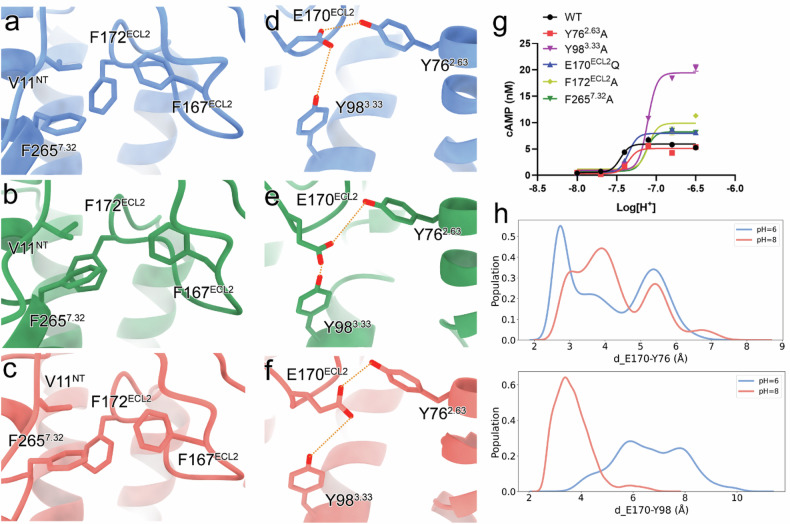
Involvement of neutral phenylalanine and tyrosine residues in proton sensing. **a**–**c** Detailed interactions of phenylalanine residues in GPR4 complexes. The GPR4–G_s_ complexes are colored blue (pH 6.5) and green (pH 7.4), respectively. The GPR4–G_q_ complex at pH 7.4 is colored salmon. **d**–**f** Detailed interactions of tyrosine residues in GPR4 complexes. Polar interactions are marked by orange dashed lines. **g** Effects of mutations on GPR4-induced cAMP accumulation. Colors are shown as indicated. The original data are provided in Supplementary Fig. S9. Values are shown as means ± SEM from three independent experiments. **h** Conformation distribution under pH 6.0 and pH 8.0. The side-chain minimal distance distributions of E170^ECL2^–Y76^2.63^ (upper) and E170^ECL2^–Y98^3.33^ (lower) are shown.

Beyond canonical proton-sensing residues, our analyses reveal crucial contributions from neutral phenylalanine and tyrosine residues (Fig. 4). The hydrophobic packing of V11^NT^, F167^ECL2^, F172^ECL2^, and F265^7.32^ facilitates ECL2 conformational shift toward the N-terminus, enabling coordinated proton sensing by polar residues (Fig. 4a–c). The phenolic hydroxyl groups of Y76^2.63^ and Y98^3.33^ stabilize E170^ECL2^, a key component of proton-sensing networks (Figs. 2i, 3c, 4d–f). Alanine mutation analysis of Y76^2.63^, Y98^3.33^, and F265^7.32^, supported by simulation results, confirmed the functional significance of these interactions (Figs. 2j, 4g, h; Supplementary Fig. S9).

### Mechanism of proton-induced activation of GPR4

**Fig. 5:**
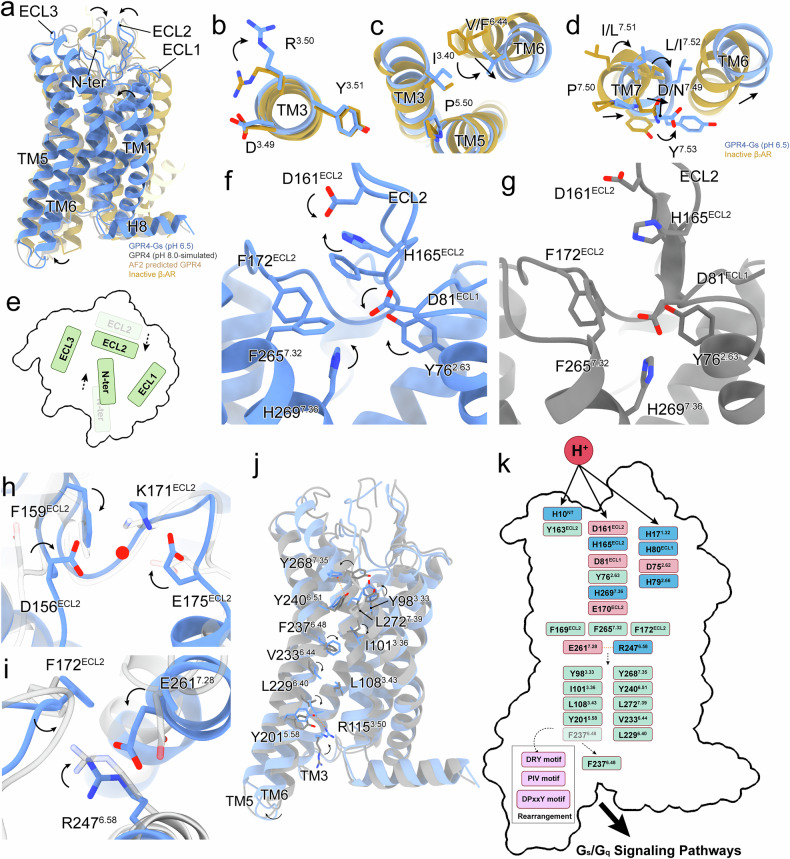
Proton-induced activation state of GPR4. **a** Overall structural comparison of the experimentally obtained GPR4–G_s_ complex at pH 6.5, the AlphaFold2 (AF2)-predicted GPR4 structure, the inactive structure of β_2_AR (PDB: 2RH1), and the simulated GPR4 at pH 8.0. **b**–**d** The conformational changes of classical motifs in GPR4, including DRY (**b**), PIV (**c**), and DPxxY (**d**) motifs, compared with the inactive β_2_AR. **e** Conformational changes of ECD upon activation. N-terminus and ECLs assemble to transduce activation signals. The transparent ones refer to their original positions and the normal ones refer to their positions after activation. The black dashed arrows indicate displacement directions. **f**, **g** Detailed conformational changes of residues related to activation of GPR4–G_s_ at pH 6.5 compared to the simulated GPR4 at pH 8.0. The former is colored in blue, and the latter is colored in gray. **h**, **i** Structural comparisons between the active and the inactive (simulated) GPR4. **j** The potential propagation path for signal transduction. Related residues are highlighted. Black arrows represent the movements of the receptor and specific residues. **k** Schematic diagram showing the proton-induced activation mechanism of GPR4. Direct interactions induced by protons are displayed by black arrows, and black dashed arrows indicate the connection of residues and conformational rearrangements of residues.

To decipher the molecular basis of pH-dependent GPR4 activation, we performed comprehensive structural comparisons among the active GPR4–G_s_ complex at pH 6.5, inactive β_2_AR structure (PDB: 2RH1), AlphaFold2-predicted GPR4 model, and simulated GPR4 at pH 8.0 (Fig. 5a).

GPR4 exhibits characteristic GPCR activation features through rearrangements of conserved motifs (D^3.49^R^3.50^Y^3.51^/P^5.50^I^3.40^V^6.44^/D^7.49^P^7.50^xxY^7.53^) (Fig. 5b–d). Notably, GPR4 contains a unique DPxxY motif instead of the canonical NPxxY motif found in most class A GPCRs. This is a feature shared with other pH-sensing receptors, GPR65 and GPR68 (Supplementary Fig. S7). This distinctive motif appears crucial for pH responsiveness, consistent with previous findings that protonation states of D^2.50^ and D^7.49^ critically influence GPR4 activation^26^.

Our structural analysis reveals that GPR4 activation initiates through significant conformational changes in ECD. The N-terminus and ECLs undergo distinctive displacement and rotation, with activation signals propagating from the N-terminus through ECL2 (Fig. 5a, e). The C9^NT^–C258^7.25^ disulfide bond serves as a critical anchor for the N-terminus, establishing a fixed reference point for proton detection and ECL recruitment (Fig. 2a). Mutations of these cysteines or N-terminus deletion severely compromise GPR4 function, highlighting their essential roles (Supplementary Fig. S6).

At both pH 6.5 and pH 7.4, key histidine residues (H10^NT^, H79^2.66^, H165^ECL2^, and H269^7.36^) in ECD undergo differential protonation in response to environmental pH. This protonation acts as a molecular switch for GPR4 activation by remodeling the electrostatic landscape of ECD and triggering receptor rearrangements (Fig. 5a, e). The polar network formed by D81^ECL1^, D161^ECL2^, and H165^ECL2^ induces repacking of a hydrophobic cluster (Y76^2.63^, F167^ECL2^, F172^ECL2^, and F265^7.32^), facilitating activation signal transduction (Fig. 5f, g). Subsequently, F159^ECL2^ repositions toward a water molecule that coordinates with D156^ECL2^, K171^ECL2^ and E175^ECL2^, stabilizing ECL2 conformation (Fig. 5h). R247^6.58^ rotates toward F172^ECL2^, establishing a salt-bridge with E261^7.28^(Fig. 5i). Core tyrosine residues, including Y98^3.33^ and Y268^7.35^, rotate toward Y240^6.51^ to propagate the signal downstream, triggering downward movements of I101^3.38^, L229^6.40^, and V233^6.44^ (Fig. 5j). Simultaneously, F237^6.48^ swings against TM3, facilitating the outward movement of TM6 (Fig. 5j). These conformational changes form the ionic lock through R115^3.50^–Y201^5.58^ interaction (Fig. 5j). Combined rearrangements of DRY, PIV, and DPxxY motifs open the intracellular pocket for G protein recruitment (Fig. 5j, k). Both simulation and mutagenesis studies validate the functional significance of these residues (Supplementary Figs. S9, S12).

Our analyses reveal a sophisticated, multi-step activation mechanism (Fig. 5k). Protonation of key histidines initiates activation through ECD conformational changes, propagating through three networks involving H10^NT^, H79^2.66^, H165^ECL2^, and H269^7.36^ (Fig. 5k). While GPR4–Gα_s_ interfaces show minimal pH-dependent differences (Supplementary Fig. S13a–e), the G_q_-coupled GPR4 at pH 7.4 exhibits slight differences, including αN rotation in Gα_q_ and α5 helix displacement (Supplementary Fig. S13a–c). The GPR4–G_q_ structure shows a unique E51^2.38^–D114^3.49^–Y358 polar network and modified ICL2–Gα interactions (Supplementary Fig. S13d–f). This suggests that proton-induced activation of GPR4 is largely driven by external conformational shifts, rather than extensive alterations in the intracellular domain. This intricate mechanism provides a comprehensive molecular framework for understanding proton-induced activation of GPR4.

### Potential lipid regulation mechanism of GPR4

**Fig. 6:**
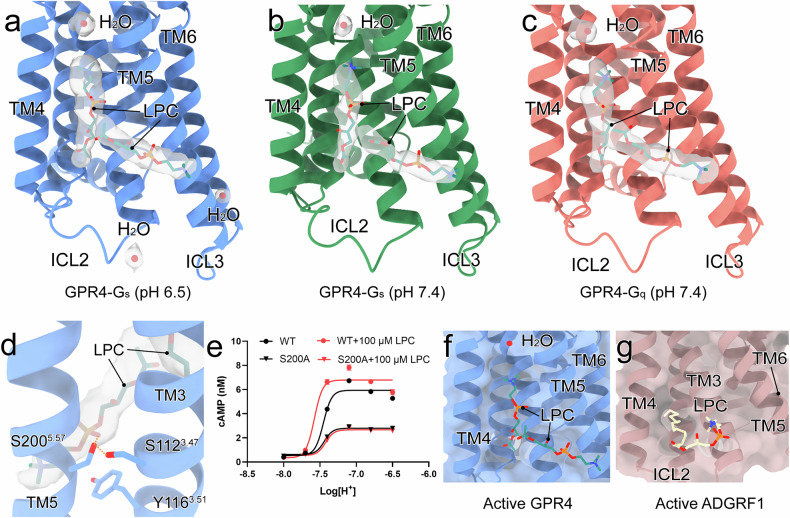
Novel lipid regulation of GPR4. **a**–**c** The overall view of LPC-binding sites around GPR4–G_s_ at pH 6.5 (**a**) and pH 7.4 (**b**), and those around GPR4–G_q_ at pH 7.4 (**c**), respectively. LPCs and water molecules are shown as sticks and red balls, respectively. **d** The engaged residues between LPCs and TM3/5 of GPR4. The polar interactions are displayed by orange dashed lines. **e** The effect of S200A mutation on LPC positive allosteric activity measured by pH-induced cAMP accumulation in GPR4 WT or mutants with or without 100 μM LPC. Values are represented as means ± SEM of three independent experiments (*n* = 3). **f**, **g** Structural superposition of GPR4 and ADHRF1 (PDB: 7WU3).

Our structural analysis revealed unexpected insights into lipid-mediated GPR4 regulation, particularly by LPC. While previous studies suggested the role of LPC in GPR4 bioactivity^57^, we identified distinct electron densities near classical allosteric sites adjacent to TM3–TM5 (Fig. 6a–c). These densities match the structural features of LPC, including its choline group, phosphate group, and short polar tail (Fig. 6a–c; Supplementary Fig. S4). This observation aligns with previous studies^35,36,57^ and our functional data showing ~30% enhancement of GPR4 activation by LPC (Figs. 1c, 6e). A conserved water molecule positioned above the putative LPC-binding site between TM4 and TM5 further defines this regulatory site (Fig. 6a–c).

The LPC-binding pocket reveals key functional interactions. S200^5.57^ forms hydrogen bonds with both the phosphate group of LPC and S112^3.47^ (Fig. 6d). S200A^5.57^ mutation significantly reduces GPR4 activation and eliminates LPC responsiveness, confirming its crucial role (Fig. 6e). Y116^3.51^ of the conserved DRY motif also interacts with LPC, suggesting a potential mechanism for allosteric modulation (Fig. 6d). Computational analyses support the role of LPC, showing that it has the lowest binding free energy among the tested lipids (Supplementary Fig. S14). This positive allosteric modulation by LPC parallels its recently reported effects on ADGRF1^51^. However, the partially overlapping binding sites between GPR4 and ADGRF1 suggest receptor-specific regulatory mechanisms (Fig. 6f, g).

## Discussion

The cell membrane functions as an environmental sensor, with proton concentration changes indicating various pathological conditions including cancer, metabolic disorders, and inflammation. Within the membrane pH-sensing components, proton-sensing GPCRs, including GPR4, contribute to cellular responses. Our study provides structural and mechanistic insights into proton sensing mechanisms of GPR4, showing relationships between receptor structure, histidine protonation, water-mediated networks, and lipid regulation.

Recent studies have highlighted the critical role of ECD’s tight association and extended polar networks formed by histidine and acidic residues^33,34^. The protonation of H^ECL2-45.47^ and H^7.36^ in non-human GPR4 activation^33^ aligns well with our findings. Notably, H10^NT^, which emerged uniquely in mammalian species^33^, plays a central role in proton recognition. Through high-resolution cryo-EM structures, functional analyses, and computational simulations, we unveil a novel proton-sensing mechanism in human GPR4. Compared with the reported proton recognition of GPR68, GPR4 employs more extensive ECD residue networks, with key histidine protonation events serving as primary triggers for pH sensing and conformational stabilization. The conserved disulfide bond (C9^NT^–C258^7.25^) acts as a molecular anchor in both GPR4 and GPR68, facilitating the characteristic inward bending of the N-terminus and TM1 critical for receptor function^34^.

Our study identifies four key proton-sensing histidines — H10^NT^, H79^2.66^, H165^ECL2^, and H269^7.36^ — whose differential protonation between pH 7.4 and pH 6.5 initiates precise conformational cascades. Similar to GPR68, GPR4 utilizes Y98^3.33^, E170^ECL2-45.52^, and H269^7.36^ to create a local polar environment facilitating signal transduction^34^. We demonstrate that the proton-induced activation mechanism of GPR4 operates through three distinct networks centered on these key histidines in ECD. These triggers coordinated tyrosine rotations in the receptor core, propagating the activation signal through transmembrane domains to enable G protein recruitment. This intricate mechanism provides a comprehensive framework for understanding proton-induced GPCR activation.

Our structural analysis also unexpectedly revealed sophisticated lipid-mediated regulation of GPR4, particularly by LPC. Functional and computational analyses identify LPC as a positive allosteric modulator, suggesting an intricate interplay between lipid binding and histidine protonation that warrants further investigation. These findings have profound implications for both basic research and therapeutic development. The high expression and involvement of GPR4 in inflammation, angiogenesis, respiratory disorders, renal dysfunction, and cancer^10,44,46^ position it as a promising therapeutic target. Our structural insights provide a foundation for structure-based drug design, as exemplified by existing antagonists like NE52-QQ57 and antagonist 3b^43–46^. Future drug development could exploit the unique histidine-mediated pH-sensing mechanism, potentially through compounds that modulate histidine protonation states or their downstream effects.

In summary, our study establishes a molecular framework for the proton sensing and activation mechanisms of GPR4, showing interactions between water-mediated networks, histidine protonation, and lipid regulation. These findings may contribute to understanding pH-related pathologies and developing targeted therapeutics. Combining structural information with computational approaches could support the development of GPR4-targeted compounds for conditions involving pH dysregulation.

## Materials and methods

### Cells

Human embryonic kidney (HEK) 293 cells were obtained from ATCC (Manassas, VA, USA). High Five (Hi5) cells were purchased from Invitrogen.

### Construct

The WT human GPR4 construct was cloned into the pFastBac^TM^ 1 vector (Thermo Fisher Scientific) with the N-terminal haemagglutinin signal peptide (HA) followed by a Flag tag and a 10× His tag. To enhance surface expression of GPR4, cytochrome b_562_RIL (BRIL)^58^ followed by a tobacco etch virus (TEV) protease site was inserted into the N-terminus of GPR4. In order to further strengthen the coupling stability of GPR4 and G protein subunits, NanoBiT strategy is applied. In detail, LgBiT fragment was fused to the C-terminus of GPCR with an optimized glycine-serine (GS) linker (GSSGGGGSGGGGSSG). Engineered Gα_s_ and Gα_q_ were used to improve the stability of GPR4 complexes. Gα_is_ is modified based on human Gα_s_ by replacing αN (MGCLGNSKTEDQRNEEKAQREANKK) with corresponding sequences of Gα_i_ (MGCTLSAEDKAAVERSKM) to facilitate scFv16 binding. Gα_isq_ is modified based on the miniGα_s_ scaffold and its N-terminus is replaced by corresponding sequences of Gα_i_1 (MGCTLSAEDKAAVERSKM). Human Gβ1 fused with a C-terminal 15-amino acid polypeptide linker (GSSGGGGSGGGGSSG) followed by a HiBiT and Gγ2 were cloned into the pFastBac^TM^ 1 vector, respectively. For cell-based functional assays in HEK293 cells, the WT *GPR4* gene was subcloned into the pcDNA3.0 vector with the addition of an N-terminal HA tag. All the mutants used for functional studies were generated by QuickChange PCR and verified by DNA sequencing.

### Expression and purification of nanobody-35 (Nb35)

Nb35 was expressed in *Escherichia coli* BL21 cells, and the cultured cells were grown in TB medium with 100 μg/mL ampicillin, 2 mM MgCl_2_, 0.1% glucose at 37 °C for 2.5 h until an optical density of 0.7–1.2 at 600 nm was reached. Then the culture was induced with 1 mM IPTG at 37 °C for 4–5 h, and the cells were collected and frozen at −80 °C. Nb35 was purified by nickel affinity chromatography, followed by size-exclusion chromatography using a HiLoad 16/600 Superdex 75 column or followed by overnight dialysis against 20 mM HEPES, pH 7.4, 100 mM NaCl, 10% glycerol. The Nb35 protein was verified by SDS-polyacrylamide gel electrophoresis and stored at –80 °C.

### Expression of GPR4–G protein complexes

Hi5 cells were infected at a cell-density of 3.0 × 10^6^ cells per milliliter; five separate baculoviruses (GPR4, engineered Gα_s_ or Gα_q_, Gβ1-HiBiT, Gγ2, and scFv16) were co-added at a rational ratio of 1:1:1:1:1 into the insect cells. After culturing for 48 h at 27 °C, the cells were harvested by centrifugation. Then cell pellets were collected and stored at –80 °C.

### GPR4–G_s_ and GPR4–G_q_ complex formation and purification

Based on the pH-dependent activation property of GPR4, we prepared two distinct buffer systems by adjusting the types and concentrations of buffer salts to maintain stable pH conditions. For GPR4–G_s_ complex in acidic condition, cell pellets were resuspended and lysed in buffer containing 20 mM 2-Morpholinoethanesulphonic acid (MES, pH 6.5), 100 mM NaCl, 5 mM CaCl_2_ and 5 mM MgCl_2_, supplemented with EDTA-free complete protease inhibitor cock (APExBIO) and apyrase (25 mU/mL, Sigma). Complex formation was initiated during the resuspension step, and the suspension was incubated for 1 h at room temperature. Then, the supernatant was removed by centrifugation at 65,000× *g* for 40 min and the pellet was resuspended. Subsequently, 0.5% (w/v) lauryl maltose neopentyl glycol (LMNG, Anatrace) supplemented with 0.1% (w/v) cholesteryl hemisuccinate (CHS, Anatrace) was added to solubilize GPR4 complex and extract it from the membrane for 3 h at 4 °C. Insoluble material was then removed by centrifugation at 65,000× *g* for 40 min. The solubilized GPR4–G_s_ complex was incubated with Talon affinity resin overnight with 10 mM imidazole (pH 6.5) to avoid impurity binding. The resin was collected and washed with 20 column volumes of 20 mM MES (pH 6.5), 100 mM NaCl, and a concentration gradient (12/15/18 mM) of imidazole (pH 6.5) and detergents (LMNG, GDN, and CHS). The complex was eluted with the buffer containing 20 mM MES (pH 6.5), 100 mM NaCl, 250 mM imidazole, 0.01% (w/v) LMNG and 0.002% (w/v) CHS, 0.005% (w/v) GDN and 0.001% (w/v) CHS. Finally, the complex was concentrated using a 15 mL 100 kDa cut-off Amicon Ultra Centrifugal Filter (Millipore), and Nb35 was added at a mole ratio of 1.5:1 incubating with GPR4 complex. The complex sample was then loaded onto a size exclusion chromatography on a Superose 6 Increase 10/300GL column (GE Healthcare) equilibrated with the buffer containing 20 mM MES (pH 6.5), 100 mM NaCl, 0.00075% (w/v) LMNG and 0.00015% (w/v) CHS, 0.00025% (w/v) GDN and 0.00005% (w/v) CHS. The peak fractions of GPR4–G_s_ complex were collected and concentrated to 11.6 mg/mL using a 500 μL 100 kDa cut-off Amicon Ultra Centrifugal Filter (Millipore) for cryo-EM grid preparation. The purification process of GPR4–G_s_ and GPR4–G_q_ complexes at pH 7.4 is similar except for the substitution of 20 mM MES with 20 mM HEPES. The final purified samples of GPR4–G_S_ and GPR4–G_q_ at pH 7.4 were concentrated to 12.0 mg/mL and 5.2 mg/mL, respectively.

### Cryo-EM grid preparation and data collection

The purified samples of GPR4–G_s_ and GPR4–G_q_ complexes were applied onto holey carbon grids (Au300, R1.2/1.3, Quantifoil), which were glow-discharged at 25 mA for 50 s using PELCO easiGlow. Excess samples were blotted for 2 s with Ted Pella filter papers (catalog number: 47000-100) under 100% humidity at 4 °C. Afterward, the grids were vitrified by plunging into liquid ethane using a Vitrobot Mark IV (Thermo Fisher Scientific). For GPR4–G_s_ (pH 7.4) complex, cryo-EM data collection was performed on a Titan Krios G4 equipped with a Gatan K3 direct electron detector at 300 kV with a magnification of 105,000×, corresponding to a pixel size 0.824 Å. Image acquisition was performed with EPU Software (Thermo Fisher Scientific, Eindhoven, Netherlands). A total of 3751 movies were obtained at a total dose of 50 e^–^Å^–2^ over 2.5-s exposure. For GPR4–G_s_ (pH 6.5) and GPR4–G_q_ (pH 7.4) complexes, cryo-EM data collection was performed on a Titan Krios G4 equipped with a Gatan Quantum-LS Energy Filter and a Falcon 4 direct electron detector at 300 kV with a magnification of 165,000×, corresponding to a pixel size 0.73 Å. Image acquisition was performed with EPU Software. A total of 6696 and 7848 movies were obtained at a total dose of 50 e^–^Å^–2^ over 3-s exposure, respectively. All movies were collected at the Advanced Center for Electron Microscopy at Shanghai Institute of Materia Medica, Chinese Academy of Sciences.

### Cryo-EM data processing

All dose-fractionated image stacks were subjected to beam-induced motion correction by RELION4.0^59^. The defocus parameters were estimated by CTFFIND4.1^60^. The following data processing was performed using RELION4.0 and CryoSPARC4.4.1, respectively.

For the GPR4–G_s_ complex at pH 6.5, data processing was performed in RELION4.0. Particle selection yielded 2,798,739 particles, which were subjected to reference-free 2D classification. The map of LY3154207–DRD1–G_s_ (PDB: 7CKZ)^61^ low-pass-filtered to 40 Å was used as an initial reference model for 3D classification, and 2,312,673 particles were selected for further processing. Then, multiple rounds of 3D classifications produced one high-quality subset accounting for 596,409 particles. These particles were subsequently subjected to 3D refinement, post-processing, and deepEMhancer^62^, which generated a map with an indicated global resolution of 2.36 Å at a Fourier shell correlation (FSC) of 0.143.

For the GPR4–G_s_ complex at pH 7.4, data processing was performed in CryoSPARC4.4.1. Particle selection yielded 3,349,414 particles, which were subjected to reference-free 2D classification. After rounds of 2D classification, 206,260 low-quality particles were subjected to ab-initio reconstruction and produced three distinct density maps. Then, we imported the map of the solved GPR4–G_s_ complex (pH 6.5) and combined them as input 3D references for hetero refinement. One obviously high-quality subset of 626,376 particles was chosen for further optimization, including global/local CTF refinement, homo/non-uniform refinement, local refinement, and deepEMhancer. A map with an indicated global resolution of 2.59 Å at an FSC of 0.143 was generated.

For the GPR4–G_q_ complex at pH 7.4, data processing was performed in CryoSPARC4.4.1. Particle selection yielded 4,555,181 particles, which were subjected to reference-free 2D classification. After rounds of 2D classification, 245,566 low-quality particles were subjected to ab-initio reconstruction and produced three distinct density maps. Then, we imported the map of the solved GPR4–G_s_ complex (pH 6.5) and combined them as input 3D references for hetero refinement. One obviously high-quality subset of 550,055 particles was chosen for further optimization, including global/local CTF refinement, homo/non-uniform refinement, local refinement, and deepEMhancer. A map with an indicated global resolution of 2.55 Å at an FSC of 0.143 was generated.

### MD simulation

The simulation systems were derived from the GPR4–Gs protein complex at pH 6.5, with G proteins removed prior to simulations. Acetyl (ACE) and N-methyl (NME) groups were added using PyMOL as previously described^63^. The GPR4 was embedded into a 75 Å × 75 Å POPC lipid bilayer using packmol-memgen software^64^, surrounded by a 15 Å aqueous layer. Ionic strength was adjusted to 0.15 mol/L NaCl, with additional counterions. We utilized the FF19SB, Lipid21, and GAFF2 force fields for amino acids, lipids, and ligands, respectively^65–67^. Systems underwent a minimization and a six-step equilibration process following the CpHMD prep protocol (https://gitlab.com/shenlab-amber-cphmd/cphmd-prep). Three independent 500-ns production runs were conducted for each system using pmemd.cuda in Amber22^68^ under the NPT ensemble at 300 K and 1 atm. pH values of 6 and 8 were maintained in all-atom mode. Long-range electrostatic interactions were managed using the Particle Mesh Ewald method, while short-range electrostatic and van der Waals interactions were handled with a 12 Å cutoff, transitioning smoothly between 10 Å and 12 Å. SHAKE was applied to constrain the bonds containing hydrogens, permitting the timestep of 2 fs. Minimal distances were calculated using the “nativecontact” command in CPPTRAJ^69^.

### Binding free energy calculation

Molecular Mechanics/Generalized Born Surface Area (MM/GBSA) was applied for binding free energy estimation. For MM/GBSA calculations, analogs of LPC were generated based on the GPR4 complex at pH 6.5 using the builder module in PyMOL. These complexes were subsequently prepared using the Protein Preparation Wizard in Schrödinger’s Maestro. During this process, bond orders were assigned, hydrogens were added to the protein, disulfide bonds were created, and residue heteroatom states were defined using Epik at pH 6.5. Each complex was then minimized using the OPLS4 force field, applying a 3.0 Å constraint on heavy atoms. An implicit membrane was added according to the helix orientation, with a thickness of 44.5 Å. Finally, Prime MM-GBSA calculations were performed using the VSGB solvation model and the OPLS4 force field.

### p*K*_a_ calculation

The p*K*_a_ values of histidine residues were calculated using the PROPKA server available at https://www.ddl.unimi.it/vegaol/propka.html^70^. The PROPKA algorithm estimates residue-specific p*K*_a_ values based on structural and environmental factors within protein models. Input structures of the protein were prepared in PDB format. The resulting p*K*_a_ values for histidines were extracted and plotted to compare the protonation behavior of histidine residues under these conditions. No additional modifications or parameter adjustments were made to the default settings of the PROPKA server.

### Cell culture and transfection

HEK293 cells were obtained from ATCC (Manassas, VA, USA) and cultured in DMEM supplemented with 10% (v/v) FBS, 100 mg/L penicillin, and 100 mg/L streptomycin in 5% CO_2_ at 37 °C. For transient transfection, ~2.5 × 10^6^ cells were mixed with 1 µg plasmids in 200 µL transfection buffer, and electroporation was carried out with a Scientz-2C electroporation apparatus (Scientz Biotech, Ningbo, China). The experiments were carried out 24 h after transfection.

### Stimulus buffer

Experiments were carried out in a physiological salt solution (PSS) containing 130 mM NaCl, 0.9 mM NaH_2_PO_4_, 5.4 mM KCl, 0.8 mM MgSO_4_, 1.0 mM CaCl_2_, 25 mM glucose. This solution was buffered with HEPES/EPPS/MES (8 mM each; HEM-PSS), to cover a wider pH range. The pH of all solutions was adjusted using a carefully calibrated pH meter (Mettler Toledo). All data in this report are referenced to pH values measured at room temperature.

### cAMP accumulation assay

Intracellular cAMP levels were detected with an HTRF cAMP kit obtained from PerkinElmer, according to the manufacturer’s instructions. In brief, HEK293 cells transfected with GPR4 WT receptor or its mutants were seeded into 96-well culture plates at a density of 4 × 10^4^ per well and incubated for 24 h at 37 °C in 5% CO_2_. The next day, the cells were incubated under different pH conditions for 30 min in the presence of IBMX at room temperature. The reaction was terminated by adding 100 µL lysis buffer, and product was diluted appropriately. Then, 10 µL of the lysate was transferred to 384-well assay plates, followed by the addition of 10 µL lysis buffer containing ULight-anti-cAMP and Eu-cAMP tracer. After a 60-min incubation in the dark, HTRF signals were detected with an Envision 2101 plate reader (PerkinElmer, Waltham, MA). The cAMP concentrations were determined using standard curves.

### IP1 accumulation assay

Intracellular IP1 was detected with an HTRF IP1 kit (Cisbio, 621PAPEJ), according to the manufacturer’s instructions. In brief, HEK293 cells transfected with GPR4 WT receptor or its mutants were seeded into 24-well culture plates at a density of 6 × 10^5^ per well and incubated for 24 h at 37 °C in 5% CO_2_. The next day, the cells were incubated under different pH conditions for 30 min in the presence of 50 mM LiCl at room temperature. The reaction was terminated by adding 100 µL lysis buffer, followed by repeated freeze-thaw for lysis. The lysed cells were then centrifuged at 12,000 rpm for 2 min at 4 °C. After centrifugation, 10 μL of the supernatant was removed and added to a 384-well plate. Another 10 µL lysis buffer containing D2-labeled IP1 and cryptate-labeled anti-IP1 monoclonal antibody was added. After a 60-min incubation in the dark, HTRF signals were detected with an Envision 2101 plate reader (PerkinElmer, Waltham, MA).

### LPC dose curve

HEK293 cells transfected with GPR4 WT receptor were seeded into 96-well culture plates at a density of 4 × 10^4^ per well and incubated for 24 h at 37 °C in 5% CO_2_. The next day, cells were incubated with 50 µL of pH 7.4 stimulus buffer containing LPC at various concentrations for 30 min in the presence of IBMX at room temperature. The reaction was terminated by adding 100 µL lysis buffer, and the product was diluted appropriately. Another 10 µL lysis buffer containing ULight-anti-cAMP and Eu-cAMP tracer was added. After a 60-min incubation in the dark, HTRF signals were detected with an Envision 2101 plate reader (PerkinElmer, Waltham, MA). The cAMP concentrations were determined using standard curves.

## Supplementary information

Supplemental materials

## Data Availability

The cryo-EM density maps of the GPR4–G_s_ complexes at pH 6.5 and pH 7.4, and the GPR4–G_q_ complex at pH 7.4 are available in the Electron Microscopy Data Bank (EMDB) under accession numbers EMD-61370, EMD-61372, and EMD-61371, respectively. The atomic coordinates for these complexes have been deposited in the Protein Data Bank (PDB) under accession numbers 9JCO, 9JCQ, and 9JCP, respectively.
