# Supplementary material for: Molecular mechanism of pH sensing and activation in GPR4 reveals proton-mediated GPCR signaling
Source: Cell Discov. 2025 Jun 25;11:59. doi: 10.1038/s41421-025-00807-y (PMC12187918; doi:10.1038/s41421-025-00807-y)
Supplement: Supplementary file 1 — Supplemental materials [file 41421_2025_807_MOESM1_ESM.pdf]

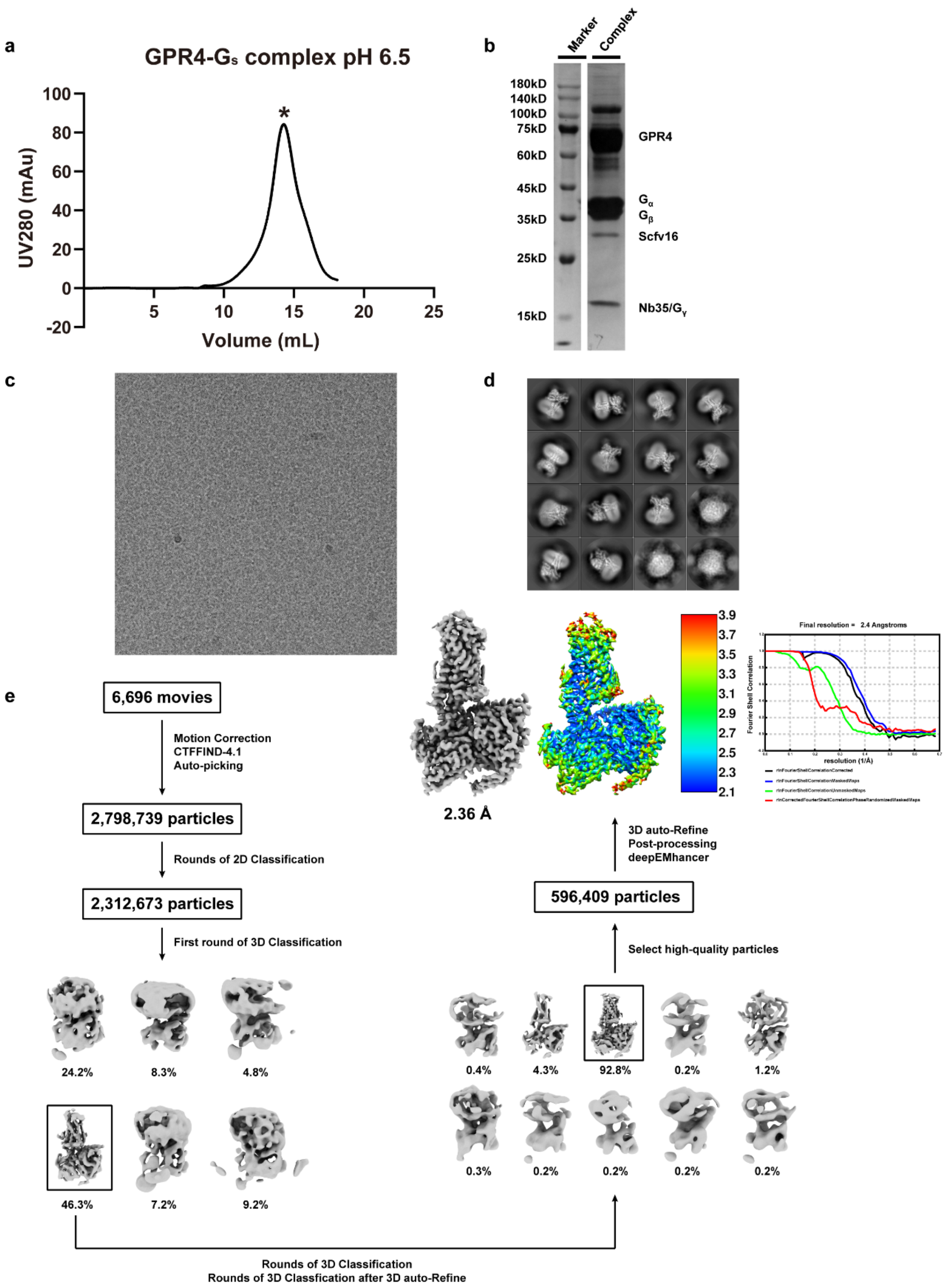

Supplementary Fig. S1 Cryo-EM data processing of GPR4-G$_s$ at pH 6.5. a-b Representative size-exclusion chromatography elution profile (**a**) and SDS-PAGE analysis (**b**) of GPR4-G$_s$ at pH 6.5. **c-d** Representative cryo-EM micrograph (**c**) and representative 2D average classes are shown (**d**). Scale bar, 50 nm. **e** Flowchart of cryo-

EM data processing, cryo-EM maps and "Gold-standard" FSC curves. Local resolution map is generated by ResMap.

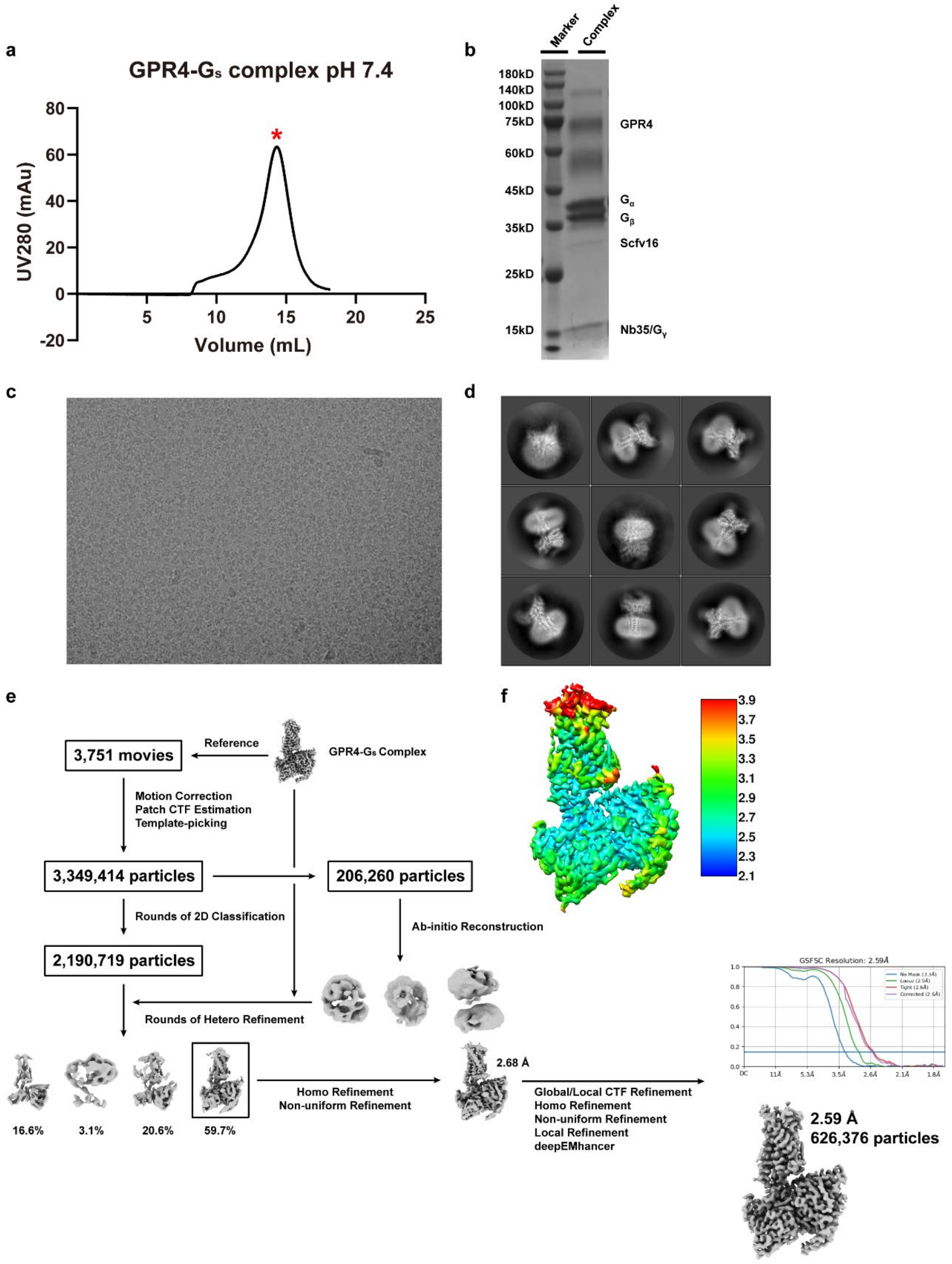

Supplementary Fig. S2 Cryo-EM data processing of GPR4-G_s at pH 7.4. a-b Representative size-exclusion chromatography elution profile (**a**) and SDS-PAGE analysis (**b**) of GPR4-G_s at pH 7.4. **c-d** Representative cryo-EM micrograph (**c**) and representative 2D average classes are shown (**d**). Scale bar, 50 nm. **e** Flowchart of cryo-EM data processing, cryo-EM maps and "Gold-standard" FSC curves. Local resolution

map is generated by ResMap.

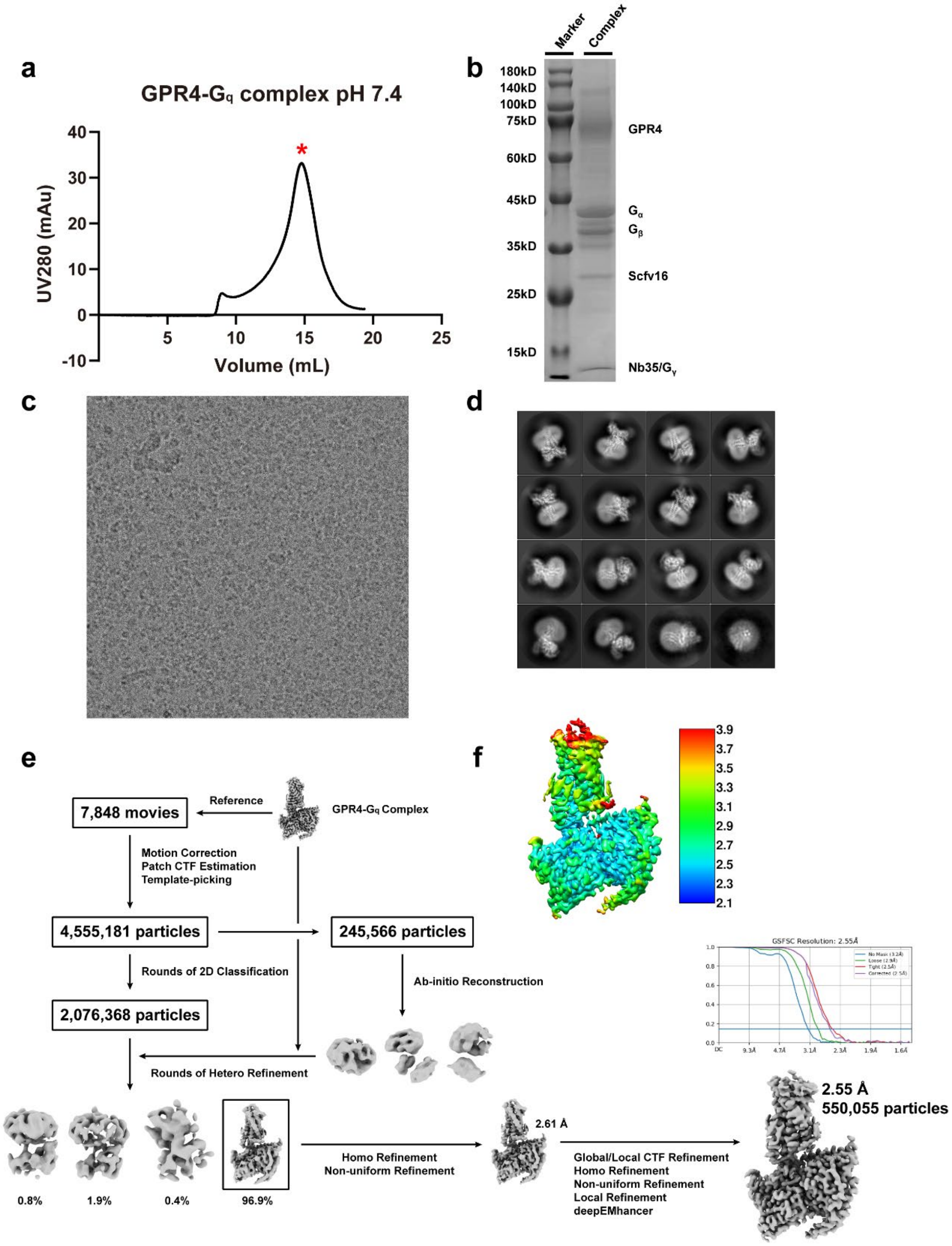

Supplementary Fig. S3 Cryo-EM data processing of GPR4-G_q at pH 7.4. a-b Representative size-exclusion chromatography elution profile (**a**) and SDS-PAGE analysis (**b**) of GPR4-G_q at pH 7.4. **c-d** Representative cryo-EM micrograph (**c**) and representative 2D average classes are shown (**d**). Scale bar, 50 nm. **e** Flowchart of cryo-EM data processing, cryo-EM maps and "Gold-standard" FSC curves. Local resolution

map is generated by ResMap.

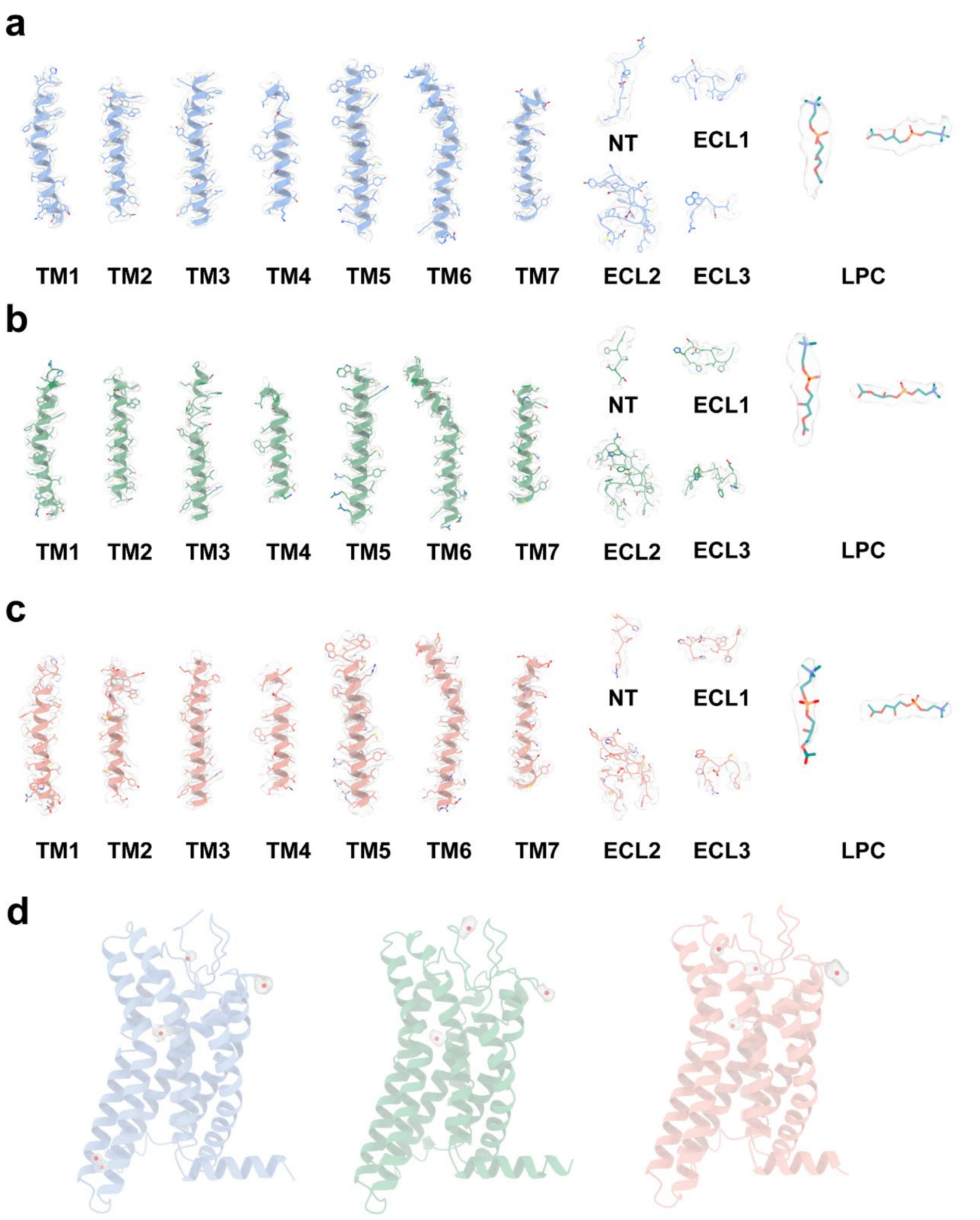

Supplementary Fig. S4 Representative cryo-EM density maps of GPR4 and lipids. Cryo-EM density maps of the seven transmembrane (TM) helices, N-terminus, ECLs, and bioactive lipids (LPC) for GPR4-G_s at pH 6.5 **(a)**, GPR4-G_s at pH 7.4 **(b)**, and GPR4-G_q at pH 7.4 **(c)**. **d** The densities of the water molecules of active GPR4 complexes. Colors are shown as indicated.

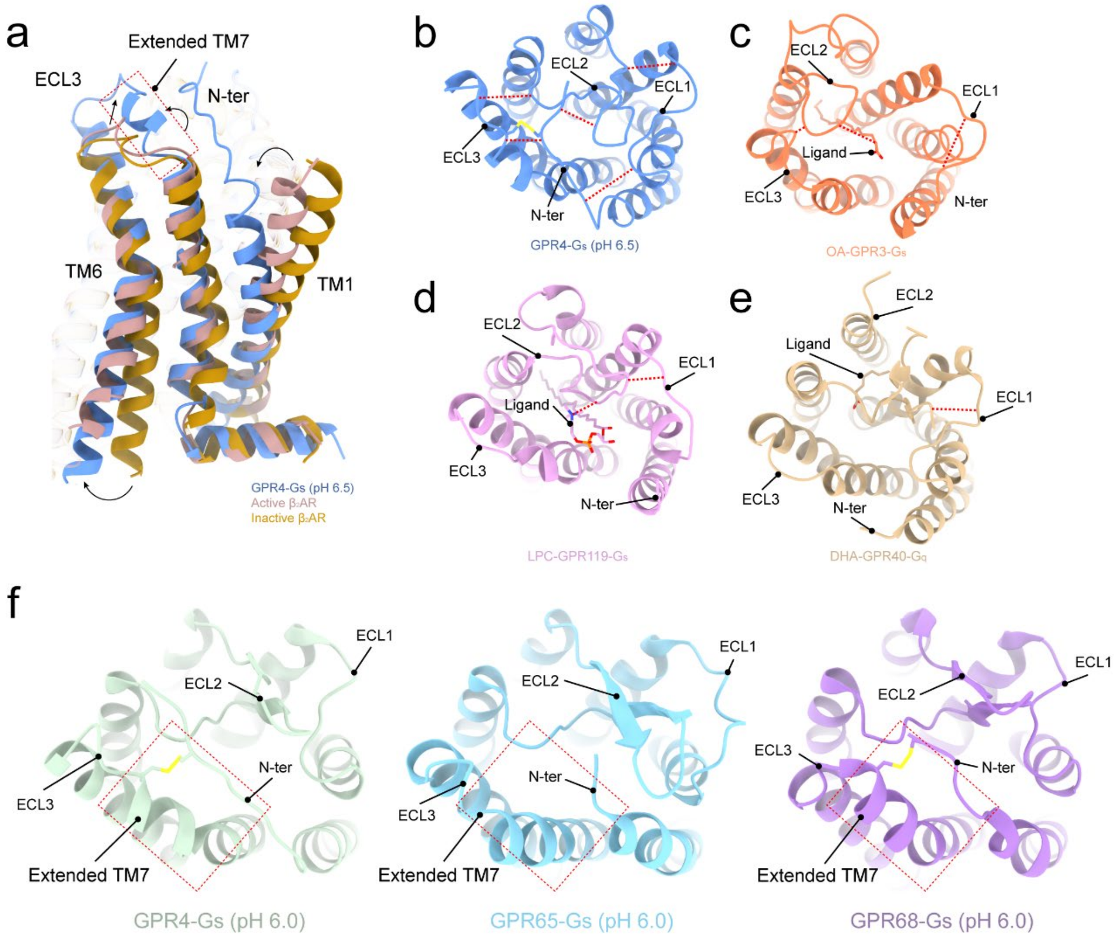

Supplementary Fig. S5 Structural comparison with representative GPCRs. a Structural comparison with active and inactive β_2AR (PDB: 7DHI/2RH1), respectively. **b-e** Distinct ECD conformations of GPR4 and lipid-liganded GPCRs. The distances are shown in red dashed lines and directions of related displacement are displayed by black arrows. **f** The top views of published proton sensing GPCRs. The dashed red rectangles display the common structural features among GPR4, GPR65, and GPR68, including a special disulfide bond and an extended TM7. Colors are shown as indicated.

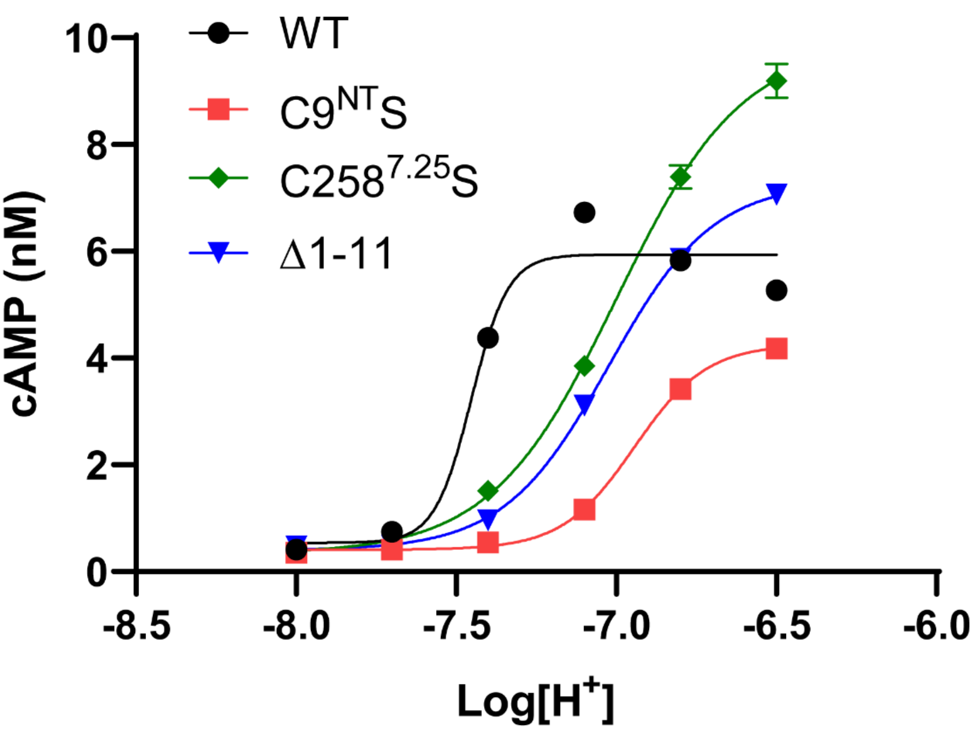

Supplementary Fig. S6 cAMP accumulation assay of pH-induced on GPR4 or mutants. Curves showing pH-dependent cAMP accumulation in cells overexpressing GPR4 or mutants. Values are represented as mean ± SEM of three independent experiments (n = 3).

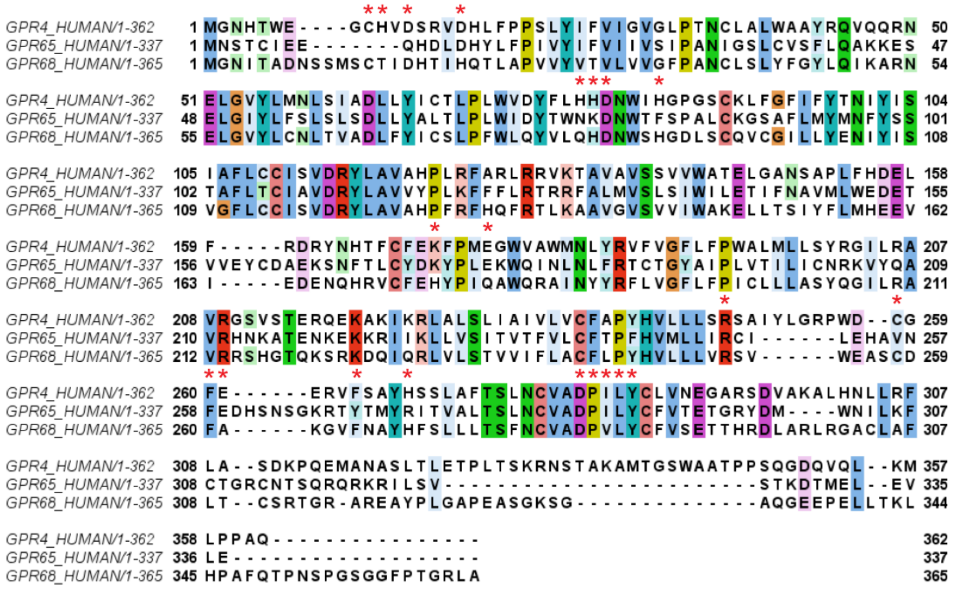

Supplementary Fig. S7 Sequence alignment of proton-sensing GPCR. Conserved sites are marked by red stars and sequence alignment is performed in Jalview by Mafft method.

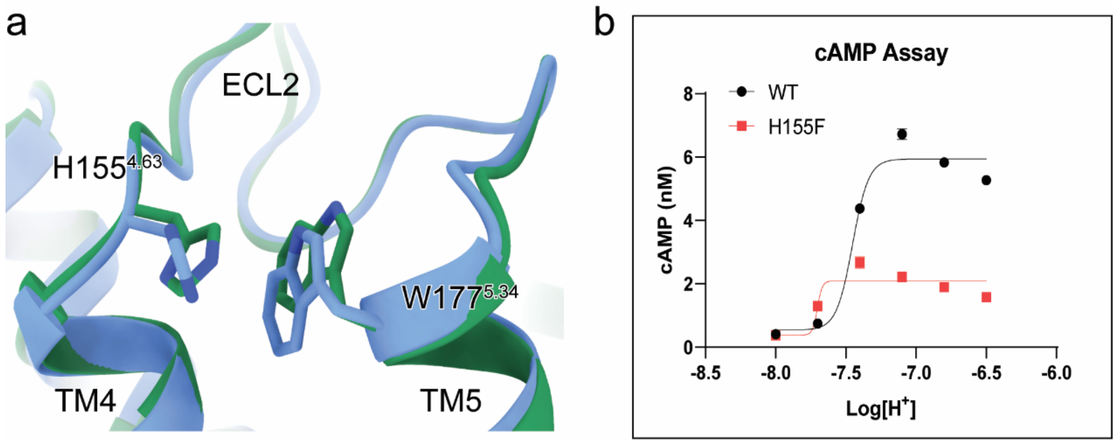

Supplementary Fig. S8 π-π stacking of H155$^{4.63}$ and W177$^{5.34}$. a The existence of the important π-π stacking between TM4 and TM5. **b** Effect of different pH on the WT and the H155F mutant of GPR4 using cAMP accumulation assay. Data shown are mean ± S.E.M. of three independent experiments.

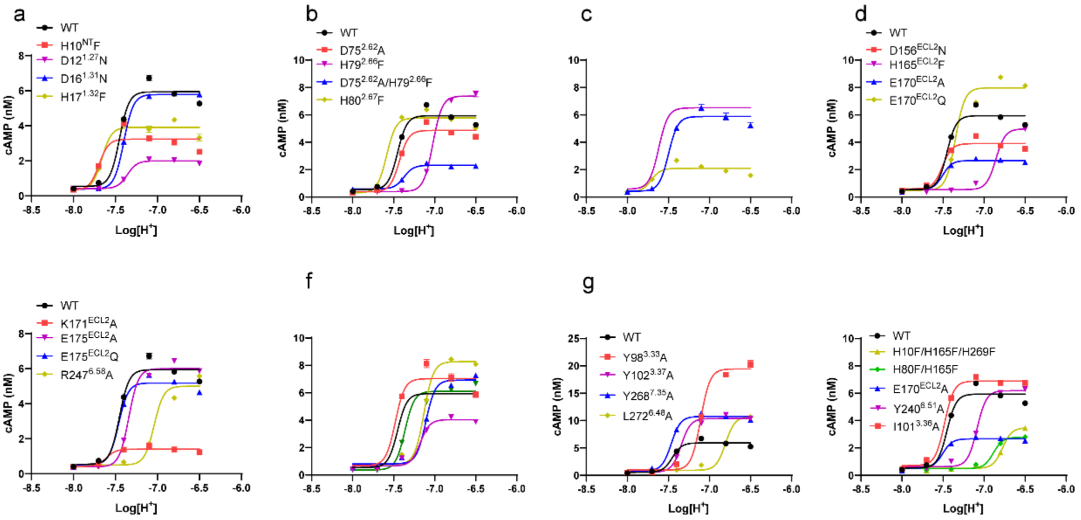

Supplementary Fig. S9 Cyclic AMP accumulation analysis for GPR4. Effects of different pH on the WT and mutants of GPR4 using cAMP accumulation assay. Data shown are mean ± S.E.M. of three independent experiments.

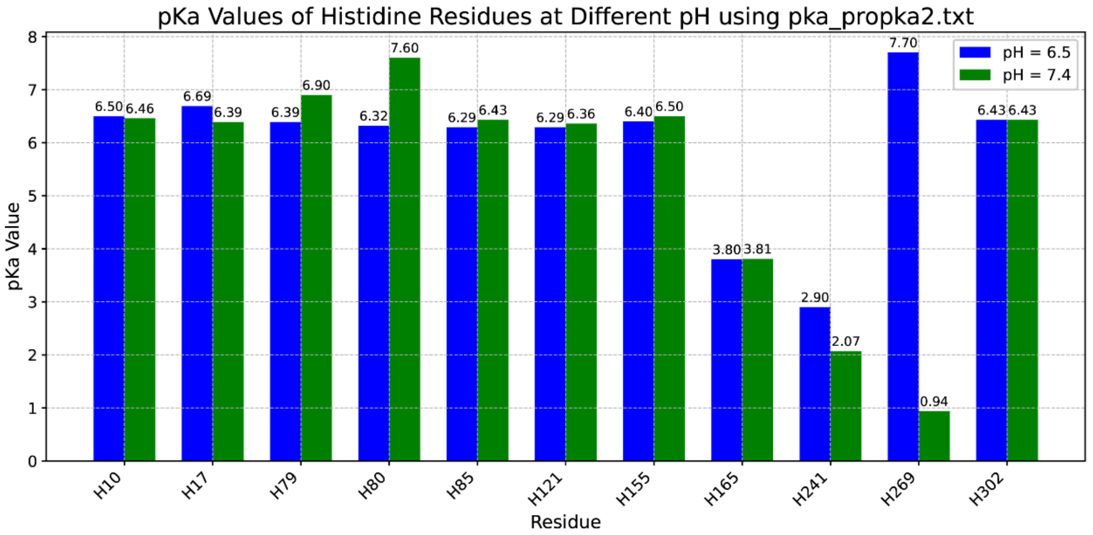

Supplementary Fig. S10 Calculations of pK_a for extracellular histidine residues by PROPKA. Residues are labeled on the x-axis, and their corresponding pK_a values are displayed above each bar.

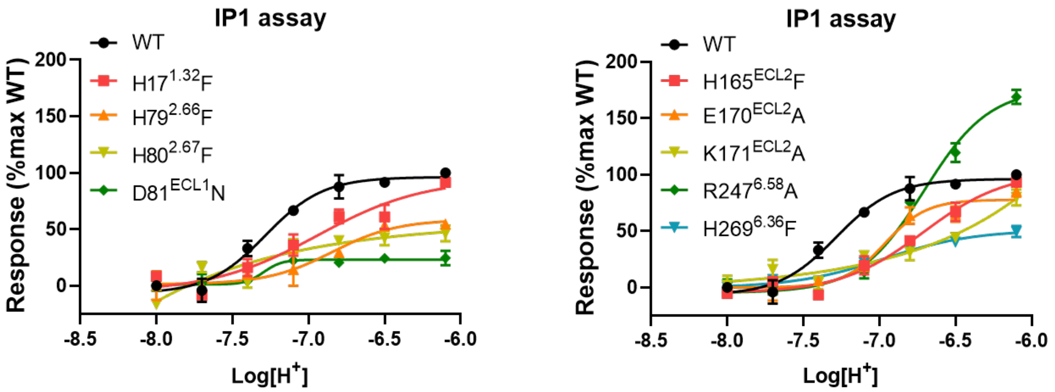

Supplementary Fig. S11 IP1 accumulation analysis for GPR4. Effects of different pH on the WT and mutants of GPR4 using IP1 accumulation assay. Data shown are mean ± S.E.M. of three independent experiments.

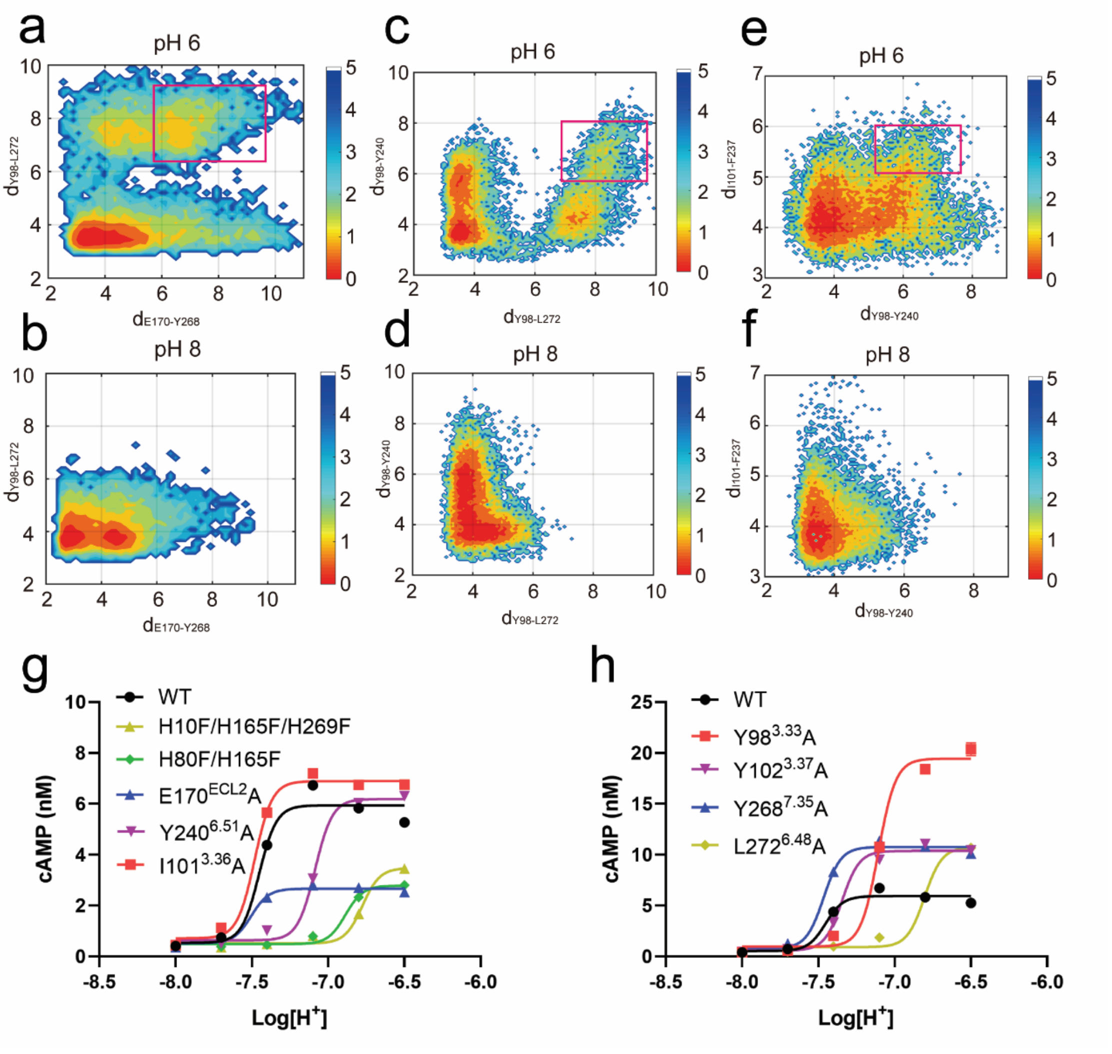

Supplementary Fig. S12 Evaluations of receptor activation mechanism exploration by molecular simulations and cAMP accumulation assay. conformation distribution under pH 6.0 and pH 8.0. a-f MD simulations of GPR4 activation. **a-b** The free energy landscape of the minimal distance between sidechain of $E170^{ECL2}$ and $Y268^{7.35}$ (X axis) and the sidechain minimal distance between $Y98^{3.33}$ and $L272^{7.39}$ (Y axis) at pH 6.0 (a) and pH 8.0 (b), respectively. **c-d** The free energy landscape of the sidechain minimal distance between $Y98^{3.33}$ and $L272^{7.39}$ (X axis) and the sidechain minimal distance between $Y98^{3.33}$ and $Y240^{6.51}$ (Y axis) at pH 6.0 (c) and pH 8.0 (d), respectively. **e-f** The free energy landscape of the minimal distance between $Y98^{3.33}$ and $Y240^{6.51}$ (X axis) and the sidechain minimal distance between $I101^{3.36}$ and $F237^{6.48}$ (Y axis) at pH 6.0 (e) and pH 8.0 (f), respectively. **g-h** Effects of different pH on the GPR4 (WT) and mutants using cAMP accumulation assay. Data shown are mean

± S.E.M. of three independent experiments.

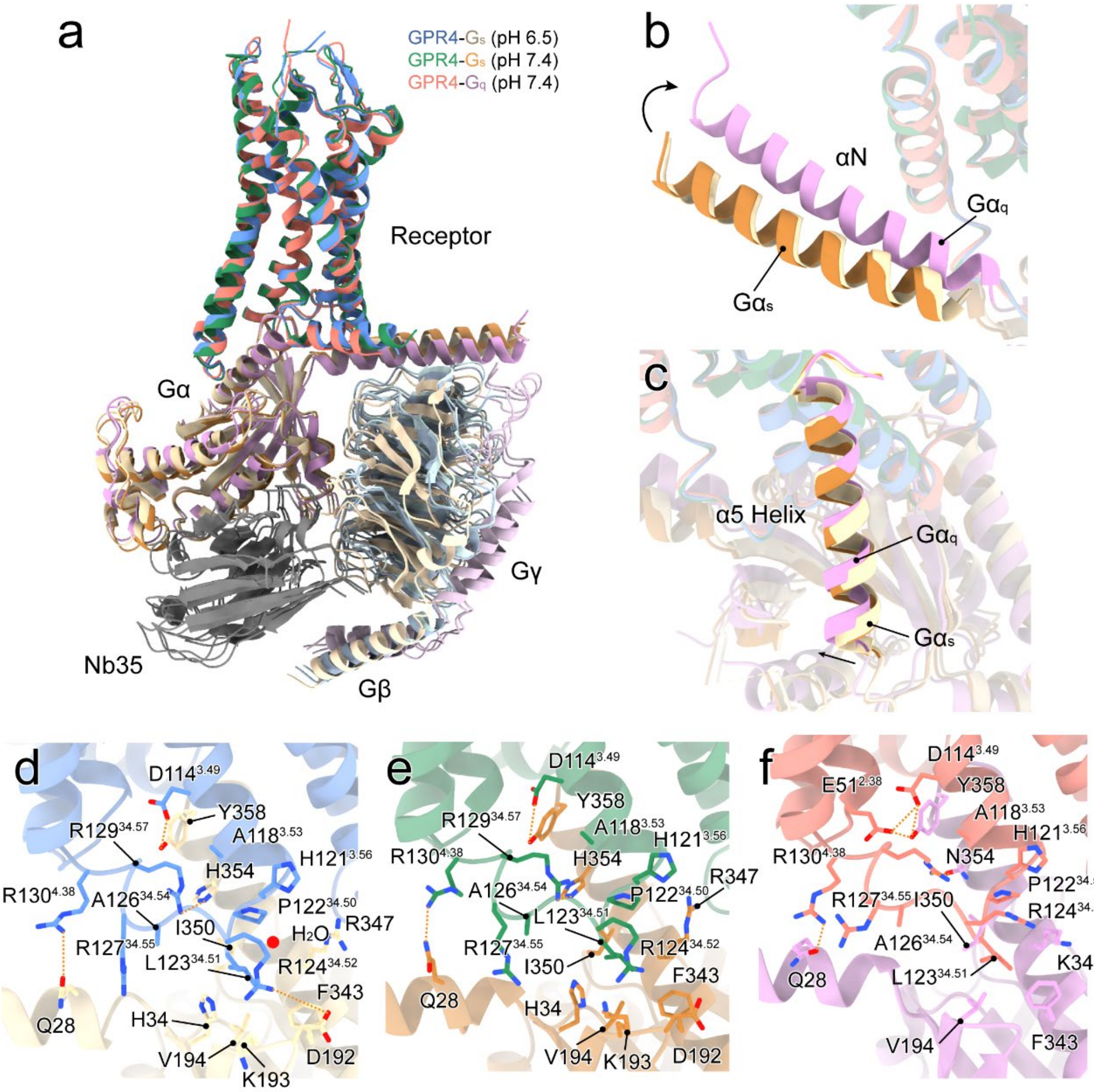

Supplementary Fig. S13 G protein interfaces between active GPR4 and $G\alpha_s$/$G\alpha_q$. **a** Overall structural comparison of GPR4 complexes. **b-c** Movement of αN and α5 Helix of $G\alpha_s$/$G\alpha_q$. The directions are shown by black arrows. **d-f** Detail interactions between GPR4 and Gα subunits. Interactions between GPR4 at different pH conditions and $G\alpha_s$ are displayed in **d** (pH 6.5) and **e** (pH 7.4), respectively. Interactions between GPR4 at pH 7.4 and $G\alpha_q$ are displayed in **f**. Colors are shown as indicated.

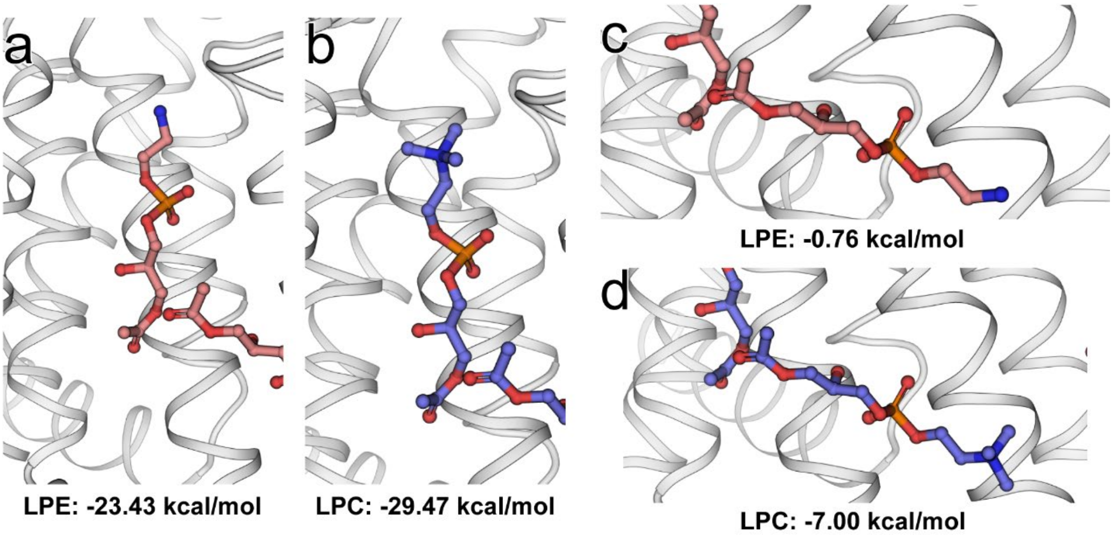

Supplementary Fig. S14 Binding free energy estimation of highly similar LPC analogues in its pocket at the top of intracellular TM4-TM5 (a-b), and at the bottom of intracellular TM4-TM5 (c-d). The binding free energy value is shown below.

Supplementary Table S1 Cryo-EM data collection, refinement and validation statistics

	GPR4-G_s (pH 6.5) (EMD-61370) (PDB: 9JCO)	GPR4-G_s (pH 7.4) (EMD-61372) (PDB:9JCQ)	GPR4-G_q (pH 7.4) (EMD-61371) (PDB: 9JCP)
**Data collection and processing**			
Magnification	105,000	64,000	105,000
Voltage (kV)	300	300	300
Electron exposure ($e^{-}/Å^2$)	50	50	50
Defocus range (μm)	-1.0~-3.0	-1.0~-3.0	-1.0~-3.0
Pixel size (Å)	0.73	0.824	0.73
Symmetry imposed	C1	C1	C1
Initial particle images (no.)	2,798,739	3,349,414	4,555,181
Final particle images (no.)	596,409	626,376	550,055
Map resolution (Å)	2.36	2.59	2.55
FSC threshold	0.143	0.143	0.143
Map resolution range (Å)	2-4	2-4	2-4
**Refinement**			
Initial model used (PDB code)			
Model resolution (Å)	2.2	2.4	2.6
FSC threshold	0.143	0.143	0.143
Model resolution range (Å)	50-2.2	50-2.4	50-2.6
Map sharpening *B* factor ($Å^2$)	-50	-60	-60
Model composition			
Non-hydrogen atoms	8431	8437	8332
Protein residues	1051	1052	1048
Ligands	2	-	-
B factors ($Å^2$)			
Protein	54.58	46.49	66.39
Ligand	58.15	41.14	47.25
Water	76.42	53.88	61.67
R.m.s. deviations			
Bond lengths (Å)	0.006	0.006	0.004
Bond angles (°)	1.034	0.723	0.617
Validation			
MolProbity score	1.55	1.31	1.30
Clashscore	7.06	5.31	3.94
Poor rotamers (%)	1.00	0.78	0.79
Ramachandran plot			
Favored (%)	97.11	97.88	97.39

Allowed (%)	2.89	2.12	2.61
Disallowed (%)	0.00	0.00	0.00
